# The Outlook for the Congress

**Published:** 1886-09

**Authors:** 


### The Outlook for the Congress.

Prof. W. F. Peck, of the Iowa State Medical University, who is now making an extended tour of Europe, writes as follows from Amsterdam to the *Jour. Am. Med. Assn.*:

"I visited Prof. Esmarch the other day in Kiel. He will come to the International Medical Congress. From what I can learn, the profession of Europe will send a large delegation. Prof. Billroth told me that he expected to attend, and Carl Braun will accompany him."

---

Frank H. Hamilton, the eminent physician and surgeon, died August 11th, at his residence, No. 43 W. 32nd street, New York. He had suffered from pulmonary hemorrhages since 1883, but had been confined to his bed only two weeks. His disease was fibroid phthisis. Dr. Hamilton was born at Wilmington, Vt., in 1813. He was graduated from the Pennsylvania University in 1833, and first settled in Auburn, N. Y. In 1844, he went to Buffalo, and with Dr. Austin Flint and Dr. J. P. White established the medical department of the Buffalo University. In 1860 he removed to Brooklyn, and after his services in the war, where he rose to be Medical Inspector of the army, he took up his residence in New York City. He was one of the founders of the Bellevue Hospital Medical College. Among the many honorary positions held by him the following may be mentioned: President of the Medical Society of the State of New York, 1855; President of the New York Pathological Society, 1866; President of the Medico-Legal Society, 1875. He was consulting surgeon of nearly all the great hospitals of this city. As a writer on medical subjects he was an accepted authority. His "Treatise on Fractures and Dislocations" is regarded in all countries as the leading work on the subject. Dr. Hamilton's boldness and success in surgical operations were remarkable. He also invented many ingenious mechanical aids to surgery. Opinions advancd by him twenty-five years ago, in regard to the treatment of bone fractures, have but lately come into general recognition. Dr. Hamilton became widely known throughout the country in 1881 as one of the physicians in attendance upon

President Garfield. He was noted for benevolence. He leaves two children, Theodore Hamilton and Mrs. Daniel Dows. His wife died a year ago.—*Cincinnati Commercial Gazette.*

